# Acute Respiratory Distress Syndrome in a Patient With Systemic Sclerosis: A Case of a Life-Threatening Complication

**DOI:** 10.7759/cureus.52003

**Published:** 2024-01-10

**Authors:** Rawan Almutairi, Dalal Alkhudair

**Affiliations:** 1 Rheumatology, Amiri Hospital, Kuwait City, KWT

**Keywords:** aspiration pneumonia, esophageal dilatation, intensive care unit, systemic sclerosis, acute respiratory distress syndrome, systemic scleroderma, diffuse cutaneous systemic sclerosis, diffuse systemic sclerosis

## Abstract

Numerous pulmonary conditions, such as aspiration pneumonia and acute respiratory distress syndrome (ARDS), may result from aspiration of gastric or oropharyngeal contents passing into the lower respiratory tract. ARDS is a type of diffuse lung injury that is distinguished by the abrupt onset of extensive pulmonary inflammation accompanied by the failure of multiple organ systems. Systemic sclerosis is an uncommon connective tissue disorder that presents with skin thickening, the etiology of which remains unknown. Esophageal luminal dilatation is observed in the distal third of the esophagus in most cases of systemic sclerosis. This dilatation is primarily attributed to the greater abundance of smooth muscle fibers in this area. Here, we present the case of a 70-year-old female patient who was diagnosed clinically with diffuse systemic sclerosis and fulfilled the 2013 European League Against Rheumatism/American College of Rheumatology classification criteria. She had esophageal dilatation, with an esophageal luminal diameter measured at the upper, middle, and lower thoracic esophagus of 2.5 cm, 2.5 cm, and 3.5 cm, respectively. The patient was admitted to the intensive care unit (ICU) due to ARDS from aspiration pneumonia. Our patient’s complicated condition at the time of ICU admission with ARDS secondary to aspiration pneumonia was primarily due to esophageal dilatation and reflux. Aggressive anti-reflux pharmacotherapy and bed elevation may be beneficial in preventing pulmonary injury caused by aspiration. Esophageal complications are common in such patients and can have a substantial impact on the prognosis and quality of life. Regular medical attention is necessary to identify and manage any potential issues.

## Introduction

Several pulmonary complications, including airway obstruction, aspiration lung abscess, aspiration pneumonia, aspiration pneumonitis, and even acute respiratory distress syndrome (ARDS) [1], may result from the aspiration of oropharyngeal or gastric contents that pass into the lower respiratory tract. ARDS is a type of diffuse lung damage characterized by acute severe inflammation caused by a dysregulated host systemic inflammatory response in the lungs. Diffuse damage to the alveolar-capillary membrane induces interstitial and alveolar edema accompanied by the formation of a protein-rich neutrophilic exudate that impairs gas exchange and decreases lung compliance. ARDS occurs frequently in cases involving severe trauma, pneumonia, sepsis, or aspiration of gastric contents, comprising around 10% of intensive care unit (ICU) patients globally [2]. Additionally, gastric aspiration is recognized as a significant direct contributor to ARDS, with around one-third of patients who present with aspiration pneumonitis progressing to ARDS, which is characterized by a more prolonged and severe course [3].

Systemic sclerosis is an uncommon connective tissue disorder with a complex and unclear cause and is primarily characterized by thickening of the skin [4]. The most prevalent manifestation, following skin disease and Raynaud phenomenon, is gastrointestinal involvement, primarily characterized by gastrointestinal dysmotility and reflux. The occurrence of esophageal reflux can be attributed to various reasons, such as impaired or absent muscular contractions in the esophagus, decreased pressure in the lower esophageal sphincter, the presence of a hiatal hernia, delayed stomach emptying, malfunction of the autonomic nerves, and the presence of sicca syndrome [5]. Reflux can cause a significant problem known as chronic microaspiration, which can result in the development of fibrosis in the lower parts of the lungs. This fibrosis can be observed on chest radiography and is similar to fibrosing alveolitis. In addition, excessive aspiration of significant volumes of fluid can result in the development of bacterial pneumonia and ARDS [6]. Here, we present a case of aspiration pneumonia in a patient with diffuse systemic sclerosis and esophageal dilation complicated by ARDS necessitating ICU admission.

## Case presentation

A 70-year-old Kuwaiti female patient presented to the emergency department with chief complaints of decreased oral intake, drowsiness, and fatigue for the last three days. Her family reported that one week before admission, she started to feel cold with rigor, fatigued, and weak. She also experienced shortness of breath when mobilizing inside the house. The family realized that, over the past few days, she slept more, became less wakeful, and was consuming less food. According to her family, she did not have syncope.

**Figure 1:**
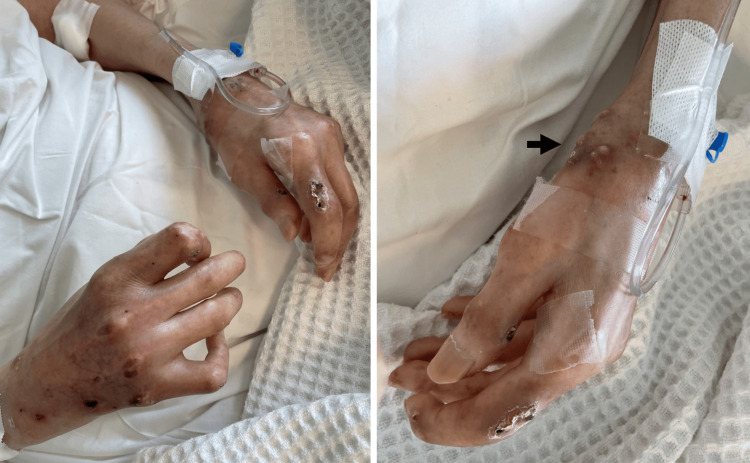
Clinical manifestations on the hands. Sclerodactyly is seen as a tightening and thickening of the finger’s skin. This causes the fingers to curl inward resulting in fixed contracture deformity of the digits due to skin thickening and forming a clawed-shaped hand. There are multiple skin ulcerations over bony prominences where the skin is contracted and tight and susceptible to trauma. The black arrow shows white chalk hard material consistent with calcium deposits indicating calcinosis cutis.

**Figure 2:**
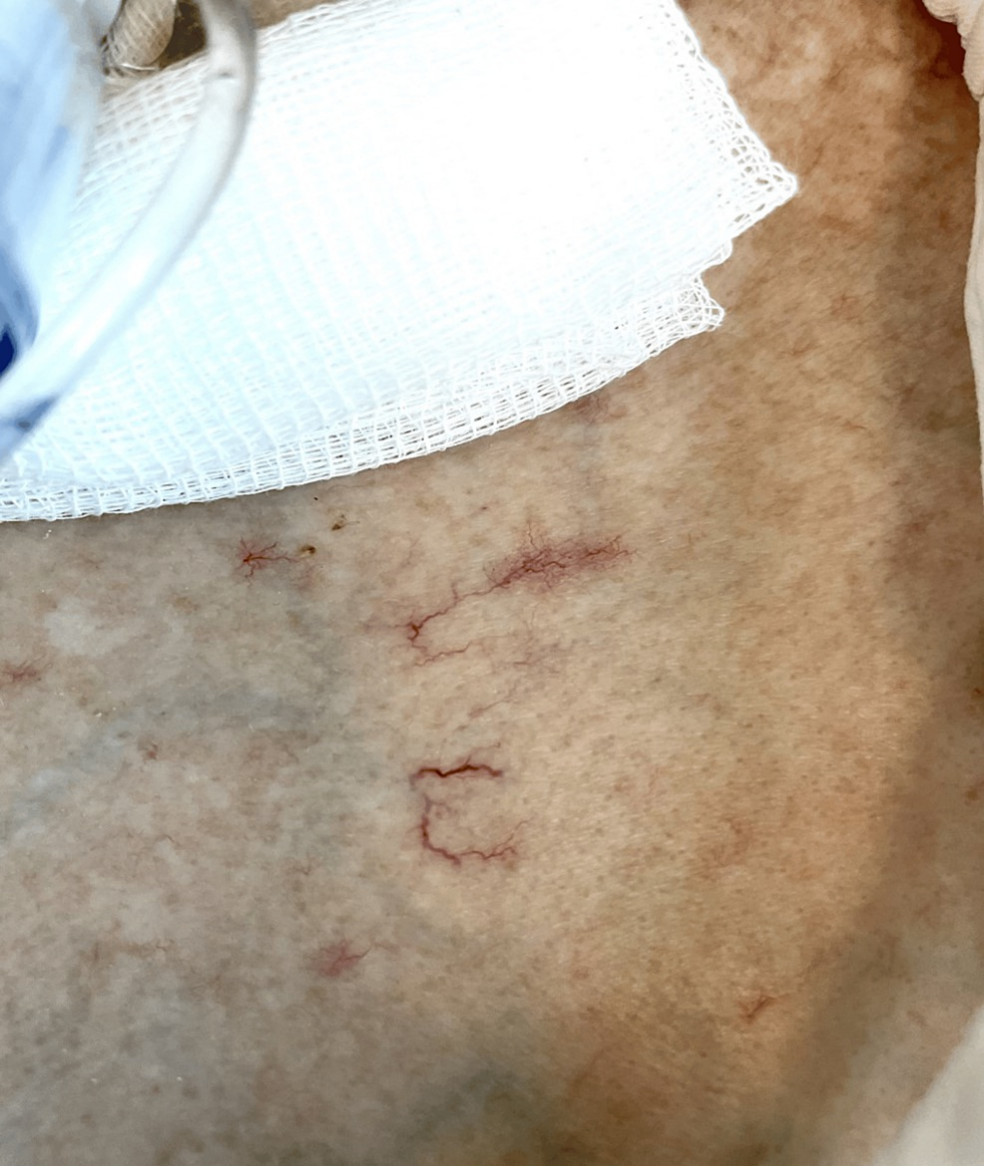
Skin manifestations on the chest. Skin hyperpigmentation and hypopigmentation on her chest, known as salt-and-pepper rash, and telangiectasia.

**Figure 3:**
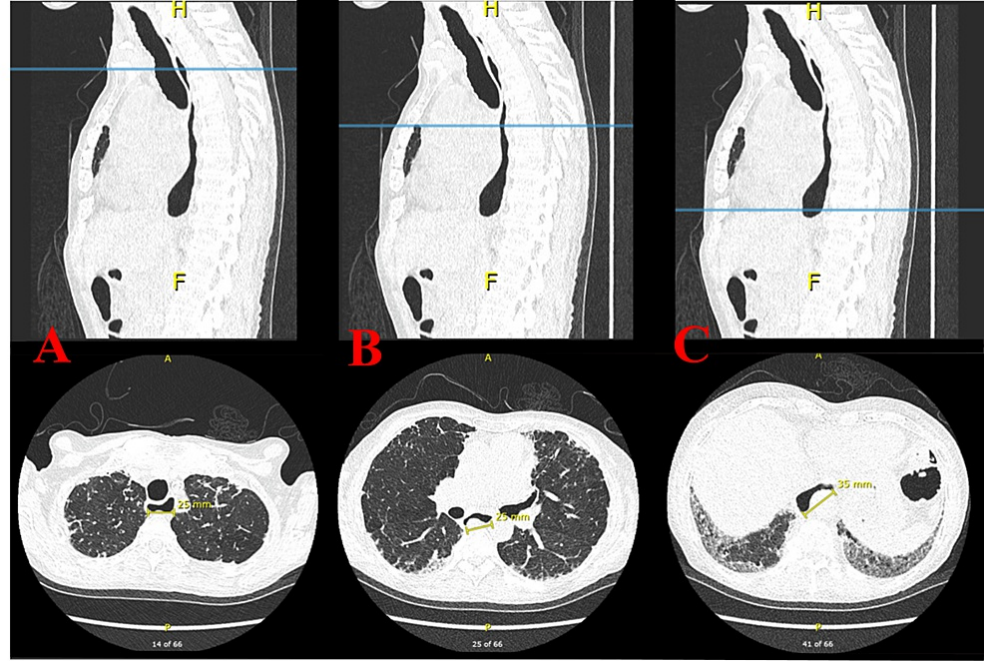
High-resolution computed tomography of the chest: sagittal and axial views. Esophageal luminal diameters measured in the upper (A), middle (B), and lower (C) thoracic esophagus were 2.5 cm, 2.5 cm, and 3.5 cm, respectively. Bilateral basilar fibrotic changes were noted. The test was done six months before the current hospital admission.

The patient was diagnosed with diffuse systemic sclerosis according to the 2013 European League Against Rheumatism/American College of Rheumatology (EULAR/ACR) classification criteria [7], complicated by interstitial lung disease, pulmonary arterial hypertension, skin thickening (Figures 1, 2), and esophageal dilation (Figure 3). An elevated pulmonary artery systolic pressure (37.21 mmHg; normal = 11-20 mm Hg) was detected on echocardiography. Immunological tests were positive for anti-nuclear antibody (titer 1:640), anti-Scl-70, and anti-SSB. Her home medications per oral included bosentan 125 mg twice daily, mycophenolate 1,000 mg twice daily, omeprazole 20 mg once daily, ramipril 5 mg once daily, bisoprolol 2.5 mg once daily, and crestor 20 mg once daily.

Upon arrival at the emergency department, she was drowsy, and her vital signs included a regular heart rate of 120 beats per minute, blood pressure of 91/46 mmHg, oxygen saturation of 85% on room air, and temperature of 37.1°C. Upon examination, the chest showed poor inspiratory effort, with right-sided coarse basal crepitations. No signs of pulmonary edema were noted. The abdomen was soft and lax on palpation, and the lower limbs were stiff, with no edema or signs of deep vein thrombosis. Skin involving the face, torso, and extremities was consistent with sclerodactyly. The patient lost the digital pulp of her fingers without digital fissures. White chalk hard material consistent with calcium deposits was identified in both hands. This patient was also noted to have areas of skin hyperpigmentation and hypopigmentation on her chest, clinically consistent with salt-and-pepper rash, and telangiectasia.

Laboratory investigations were requested, including venous blood gas analysis, which showed a pH of 7.21, pCO_2_ of 11.1 mmHg, lactate of 4 mmol/L, and HCO_3_ of 26.4 mEq/L. She was then placed on bilevel positive airway pressure, but her venous blood gas further deteriorated to a pH of 7.1, pCO_2_ of 9 mmHg, pO_2_ of 22 mmHg, and HCO_3_ of 19.3 mEq/L. She was unresponsive and was urgently intubated and mechanically ventilated. Moreover, a complete blood count showed a high level of white blood cells (29.5 × 10^9^/L) with neutrophilic predominance (95.7%), hemoglobin (110 g/dL), and platelets (257 µL). Renal function test showed high creatinine 652 µmol/L. Furthermore, her C-reactive protein level was 400 mg/L. Chest radiography revealed moderate right pleural effusion.

The patient was oliguric and resuscitated with two pints of normal saline, but her blood pressure remained low; hence, norepinephrine was started peripherally, and she was admitted to the ICU. Upon arrival at the ICU, she was started on meropenem and clindamycin empirically for aspiration pneumonia. Endotracheal tube culture grew *Streptococcus pneumoniae* sensitive to ceftriaxone; therefore, ceftriaxone was started and completed for seven days.

During her stay in the ICU, she persistently had leukocytosis of up to 30 µL. Blood film analysis was performed twice and showed leukocytosis, mostly neutrophilic, with a mild left shift of the granulocytes and a few toxic granulations. Therefore, the pan culture was repeated. Endotracheal tube culture grew *Stenotrophomonas maltophilia*, and she was started on co-trimoxazole for seven days.

**Figure 4:**
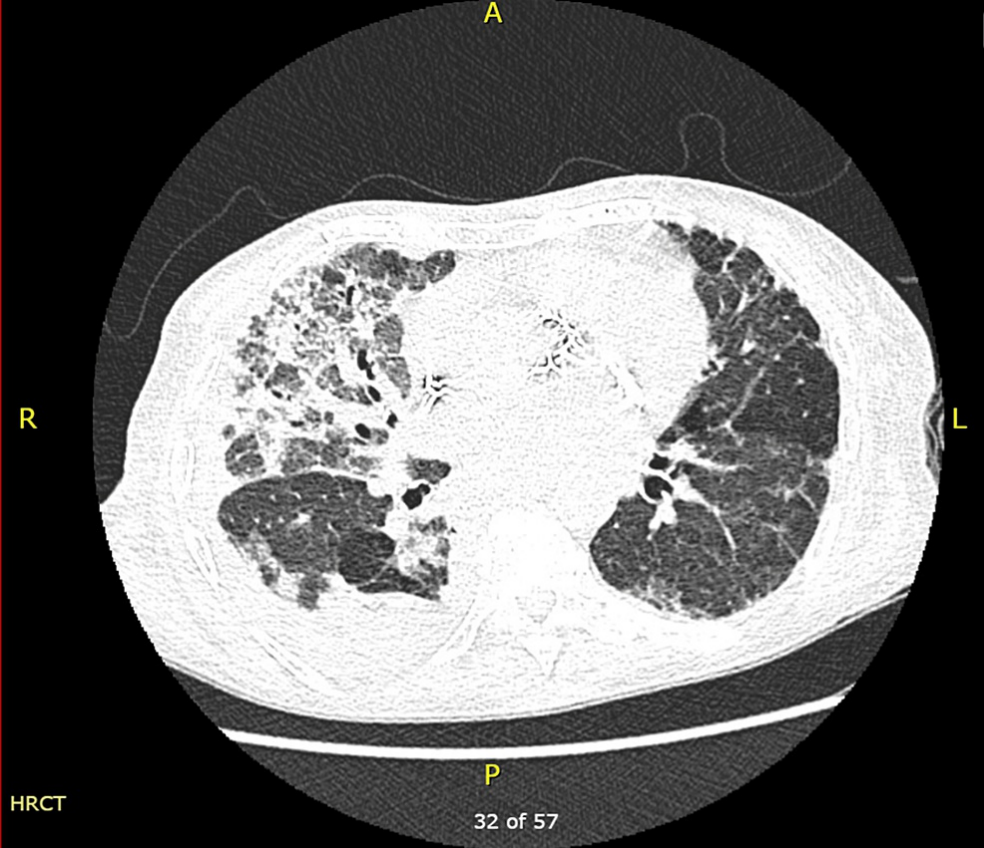
High-resolution computed tomography of the chest: axial view. Increased ground-glass opacities and bilateral patchy fibrosis in the middle lobe and more in the right lower lobe. Right middle and lower lobe segmental consolidation, and right moderate pleural infusion. The test was done during the current hospital admission.

Multiple trials of weaning from mechanical ventilation failed because the patient accumulated CO2 and became tachypnic, which was associated with hemodynamic instability. The chest CT ruled out a pulmonary embolism but detected interstitial lung disease related to systemic sclerosis, consolidation in the right middle lobe, and right pleural effusion (Figure 4). Subsequently, the patient underwent a bedside percutaneous tracheostomy and was connected to a mechanical ventilator. Feeding was performed using a nasogastric tube.

After two weeks in the ICU, the patient was discharged to the medical ward as her general condition improved. The patient was vitally stable and afebrile. Laboratory investigations, including complete blood count, C-reactive protein levels, and renal function test, were normal. However, the patient still required mechanical ventilation.

## Discussion

Systemic sclerosis is a complex disease that affects connective tissues, with the involvement of multiple organs and a wide range of clinical symptoms. However, the cause of this disease remains unknown. The annual incidence of systemic sclerosis is approximately 20 cases per million people, whereas its prevalence in the United States is over 250 patients per million individuals [4,8]. The role of genetics in influencing this disease has been a subject of research for a considerable period. Disease incidence can vary from 1.5% to 1.7% in families with a history of systemic sclerosis. The risk of having the disease is increased by 15 to 19 times in siblings and by 13 to 15 times in first-degree relatives with a family history [9,10].

The clinical and pathologic manifestations of the disease arise from three distinct processes, namely, abnormalities in the innate and adaptive immune systems which result in the production of autoantibodies and cell-mediated autoimmunity, microvascular endothelial cells and fibroproliferative vasculopathy affecting small vessels, and fibroblast dysfunction leading to excessive accumulation of collagen and other matrix components in the skin, blood vessels, and internal organs [9].

Disease symptoms range from diffuse cutaneous involvement, which involves widespread skin along with internal organ involvement, to limited cutaneous, which involves only limited skin. Table 1 lists the primary distinctions between these two forms, i.e., the degree of skin involvement, the relation of autoantibodies, and the progression of organ involvement [9,11].

**Table 1:** Different features between limited cutaneous systemic sclerosis and diffuse cutaneous systemic sclerosis. [9,11].

Features	Limited cutaneous systemic sclerosis	Diffuse cutaneous systemic sclerosis
Cutaneous	Skin thickening occurs late, limited to the distal part of the upper and lower extremities, face, neck, and upper chest. Telangiectasias and calcinosis are common. Tendon friction rub is not seen	Skin thickening occurs early and moves up to the proximal part of the extremities and trunk. Telangiectasias and calcinosis may occur late in the disease. Tendon friction rub is present
Gastrointestinal	Esophageal dysmotility is more common than small and large intestine involvement	Esophageal dysmotility is frequently seen. Small and large intestinal involvement is more common
Pulmonary	Pulmonary fibrosis is less frequent and less severe. Frequent and severe pulmonary hypertension is more common	Pulmonary fibrosis is more common and severe. Pulmonary hypertension is less frequent
Renal	Renal crisis is uncommon	Renal crisis is more frequent
Autoantibody association	Anticentromere antibodies are predominant	Anti-DNA topoisomerase I antibody (Anti-Scl-70) antibody is predominant. Anti-RNA polymerase antibody is more common

Visceral complications are the leading cause of morbidity and mortality in patients with systemic sclerosis. The most serious consequences seen in this illness include interstitial lung disease, pulmonary hypertension, pericarditis, cardiac conduction abnormalities, and renal crises [12]. Nevertheless, despite being frequently disregarded, the esophagus is the most commonly impacted internal organ, affecting up to 90% of patients [13]. Fibrous tissue replaces smooth muscle atrophy that occurs early in the progression of the disease. These alterations have the potential to result in significant dysmotility of the distal esophagus, dilatation, absence of body peristalsis, and decreased pressure on the lower esophageal sphincter. Gastroesophageal reflux accompanied by recurrent microaspiration of gastric contents may be a significant risk factor for the development of interstitial lung disease, according to the literature [14]. Esophageal luminal dilatation is observed in the distal third of the esophagus in most cases of systemic sclerosis. This dilatation is primarily attributed to the greater abundance of smooth muscle fibers in this area [15]. In one study with a sample of 20 patients with systemic sclerosis, 80% showed esophageal dilatation on high-resolution CT, ranging from 13 to 44 mm, with luminal distention detected near the lower esophageal sphincter [16]. Consistent with recent studies, esophageal dilatation was identified when the luminal diameter exceeded 10 mm [15,17,18].

Esophageal luminal diameters measured in the upper, middle, and lower thoracic esophagus were 2.5 cm, 2.5 cm, and 3.5 cm, respectively. We acknowledge that our patient’s complicated condition of ICU admission with ARDS secondary to aspiration pneumonia was primarily due to esophageal dilatation and reflux. Aggressive anti-reflux pharmacotherapy and elevation of the head of the bed may help reduce pulmonary damage caused by aspiration. It is important to note that proton pump inhibitors may interfere with drug absorption, particularly mycophenolate mofetil. At least a two-hour gap between them is essential [19].

## Conclusions

Esophageal complications are common among patients with systemic sclerosis and can have a substantial effect on their prognosis and quality of life. Our case showed an example of a life-threatening complication of systemic sclerosis that contributed to the development of ARDS in an already damaged lung due to interstitial disease. To improve patient outcomes and develop effective management strategies, it is critical to understand the various esophageal motility abnormalities and their correlation with the disease complications.
